# Knowledge, attitude and practice of pre-operative fasting in gastrointestinal surgery: Post-operative complications

**DOI:** 10.6026/973206300222235

**Published:** 2026-04-30

**Authors:** Mohammed Mushthaque Muzammil, Siddiqui R.A, Gourav Singh Shekhawat

**Affiliations:** 1 Department of Emergency Medicine, Dr Moopen's Medical College, Wayanad, Kerala, India; 2 Department of Pharmacology, NKP Salve Institute of Medical sciences & RC and LMH, Nagpur, India; 3 Department of Community Medicine, Rajasthan University of Health Science and Jai Durga College of Nursing, Rajasthan, India

**Keywords:** Pre-operative fasting, gastrointestinal surgery, patient compliance, perioperative care, post-operative outcomes, mixed-method study

## Abstract

Although there are evidence-based guidelines for the recommended approach to pre-operative fasting, there is still an ongoing pattern of extended use of pre-operative fasting ("nil by mouth after midnight") in gastrointestinal surgeries which may result in increased levels of patient discomfort as well as post-operative complications. Therefore, it is of interest to evaluate patients' knowledge, attitudes and practices (KAP), related to pre-operative fasting among 200 elective gastrointestinal surgical patients and to assess its association with post-operative outcomes. The average fasting duration was 10.8 ± 2.7 hours for solids and 8.2 ± 2.3 hours for liquids, which was significantly longer than the guideline recommendations and the patients had higher rates of nausea, dehydration and wound infections as evidenced by lower KAP scores (p < 0.05). Those patients who had a better understanding of pre-operative fasting as well as better practices of pre-operative fasting had shorter fasting periods and reduced complications after surgery. Thus, we show that a structured patient-centered approach for pre-operative fasting education and standardized means for communicating pre-operative fasting instructions lead to decreased periods of fasting and improved overall perioperative outcomes following gastrointestinal surgery.

## Background:

The pre-operative fasting period is designed to minimize the risk of pulmonary aspiration during anesthesia induction. Current clinical practice guidelines indicate that elective surgical patients should be allowed to have solids for no more than six hours and clear liquids for not less than two hours before their scheduled surgery [1]. Many medical centers use the traditional instruction for pre-operative fasting: "nothing to eat or drink after midnight." As a result, patients are often required to undergo prolonged pre-operative fasting unnecessarily [2]. Prolonged pre-operative fasting is associated with a variety of negative consequences, including dehydration, irritability, insulin resistance, electrolyte imbalance and prolonged recovery time [3]. Patients undergoing gastrointestinal surgery, who are already subjected to metabolic stress from their condition, will likely experience these effects more severely than other surgical patients [4]. Enhanced recovery after surgery (ERAS), which promotes an accelerated recovery process, is focused on decreasing the duration of fasting and resuming normal diets as quickly as possible following surgery; however, the implementation of these principles remains challenging for many health care providers [5, 6]. Patient-related issues, such as lack of knowledge, anxiety, miscommunication and use of out-dated instructions, can contribute to inadequate adherence to appropriate pre-operative fasting guidelines [7]. The limited amount of research evaluating the relationship between patients' knowledge, attitudes and actual pre-operative fasting practices and measured post-operative complications in the gastrointestinal surgical population has yet to be clearly defined [8]. For continuous improvement in perioperative patient safety, further investigation is warranted to establish how behavioral and communication issues will affect clinical outcomes. Therefore, it is of interest to understand gastrointestinal patients' knowledge, attitudes and practices concerning pre-operative fasting will facilitate examining their relationship with post-operative complications.

## Materials and Methods:

This study utilized a mixed methods approach and was conducted at a tertiary care teaching hospital over a 12-month period. Due to the nature of the procedures, all 200 participants underwent elective gastrointestinal surgeries under general anesthesia and were between the ages of 18 and 70 years old. The participants were recruited consecutively and excluded from the study if they were undergoing emergency surgery or were critically ill and were not able to give consent to participate in the research project. The Institutional Ethics Committee had given their approval for the study prior to commencing the project. Quantitative component included the use of a validated structured knowledge-attitude-practice (KAP) questionnaire to assess knowledge of recommended fasting times, understanding of aspiration risk and knowledge of fasting duration before surgery. A Likert scale was used to score responses and scores were assigned to the KAP domains based on established cut-offs of 'Adequate', 'Moderate' or 'Poor'. The study recorded hours of fasting for solids and clear fluids based on self- reporting by the patients and the surgical scheduling records when available. The qualitative component included semi-structured interviews with a purposely selected subgroup of 30 participants who represented different levels of KAP knowledge. The interviews focused on communication experiences of patients, barriers to understanding fasting instructions, emotional responses to fasting instructions and understanding of the rationale behind fasting before surgery. The interviews were audio recorded, transcribed verbatim and analysed thematically. Prospective collection of post-operative outcome data was performed through review of medical charts and included the presence or absence of nausea/vomiting, dehydration, electrolyte imbalance, wound infection and duration to resuming oral intake. The data was summarized using descriptive statistics and associations were assessed between KAP levels, fasting duration and post-operative complications using the chi-square test and Pearson correlation coefficient. A significance level of p < 0.05 was established for the statistical analysis using SPSS version 26.0.

## Results:

Two hundred participants who were scheduled to have elective gastrointestinal surgery were studied in this research. The average age of the participants was 46.3 ± 12.8 years. Of these participants, 59% were male and almost two-thirds (64%) were between the ages of 31-50 and 29% had some form of comorbidity. While 82% of participants stated that they were aware of the need to fast beforehand, only 41% could explain the physiological reason for fasting. The average length of time that participants fasted for solid food and clear liquid was 10.8 ± 2.7 hours and 8.2 ± 2.3 hours respectively; these times were longer than the recommended guidelines. In addition, approximately 74% of participants received instructions to be nil per os starting at midnight regardless of the time of their surgery. Participants with low levels of knowledge and practice received longer periods of fasting (qualitatively, interviews identified that communication barriers between healthcare providers, inconsistent instructions and emotional distress were contributing to the prolonged fasting time), leading to a greater incidence of post-operative complications (nausea, dehydration and wound infections). The results of this study may provide an opportunity for improved perioperative education by healthcare professionals. As depicted in Table 1 (see PDF), a significant percentage of participants (64%) aged between 31 and 50 years, with 59% of participants being male; comorbidities were reported by 29% of participants and it was reported that surgical exposure prior to the surgery was experienced by 38% of participants. As represented in Table 2 (see PDF), education levels were based on the following: 48% had secondary education and 63% belonged to the middle socioeconomic level. As illustrated in Table 3 (see PDF), 82% of participants were aware that they should fast prior to their surgery, however, only 29% of participants received information about fasting from their physician and 41% of participants received that information from a nurse. In Table 4 (see PDF), only 41% of participants identified aspiration prevention as the reason for fasting; also fewer than 30% of participants identified appropriate fasting time duration. As shown in Table 5 (see PDF), 64% were deemed excessive in their fasting experience, 67% reported experiencing anxiety while fasting and 74% indicated a willingness to adhere to updated guidelines. According to Table 6 (see PDF), the mean fasting time for solid food was 10.8 (± 2.7) hours and liquid food was 8.2 (± 2.3) hours, exceeding guidelines set forth, with adherence at 38% to 42%. In Table 7 (see PDF), 87% of participants were instructed to fast from midnight and 96% complied; however, only 13% received their fasting instructions based on guidelines. It can be seen in Table 8 (see PDF) that those with adequate knowledge experienced shorter fasting times than those with inadequate knowledge (p < 0.05). Table 9 (see PDF) shows that fasting longer than 10 hours for solid food or more than 8 hours for liquid food was associated with patients experiencing nausea, dizziness and infection. The data in Table 10 (see PDF) suggests that those with adequate knowledge and adherence to guidelines experienced lower complication rates post-operatively than those with inadequate knowledge and adherence (p < 0.05). There were also common themes of an inconsistent message, the anxiety induced by long periods of fasting and a preference for clearer counseling as found in the qualitative analysis presented in Table 11 (see PDF).

## Discussion:

Results from this mixed-method investigation indicate that long-term fasting prior to an operation is still a common practice among people undergoing gastrointestinal surgery despite established guidelines indicating that fasting time should be short. In addition to finding that the average time for fasting from solids and liquids has been well over recommended limits, researchers found that the majority of patients were instructed not to eat and/or drink anything after midnight regardless of what time their surgery would take place. Researchers determined that inadequate knowledge as well as inappropriate practice were both strongly linked to how long an individual had fasted and how many complications they had after surgery, illustrating the evidence-to-practice gaps that persist in the management of fasting preparation. Modern anesthesia guidelines recommend that a person fasts for at least six hours prior to surgery on food solids and for at least two hours prior to surgery on clear liquids [9, 10]. However, while individual facilities are encouraged to follow these guidelines, they often do not do so, particularly in situations where resources are limited [11]. The on-going insistence on fasting restrictions that state "nothing by mouth after midnight" provides evidence of the institutional inertia in organizations along with the lack of dissemination of newly defined guidelines for fasting in the perioperative phase of care. Additionally, barriers to implementation of newly established fasting guidelines have been documented recently in perioperative audits [4, 5]. When a person is required to fast for a long period of time before having a surgical procedure performed, they may develop dehydration, insulin resistance, an uncomfortable feeling and/or may take a longer time than expected for their gastrointestinal system to recover [12, 13]. Within this patient population, greater periods of fasting have been linked directly to various post-procedure complications such as nausea, lightheadedness, infection at the surgical site and dehydration. Due to the relationship between excessive fasting periods and increased post-operative complications, excessive fasting periods may also be a contributing factor regarding metabolic stress and recovery time due to the stress of surgery along with the nature of the surgery itself which causes many individuals to not eat in the days leading to their surgery [14]. The degree to which a patient possesses knowledge regarding fasting prior to surgery is an important consideration with this study. Even through the majority of patients understood that fasting was required prior to surgery, only about half were able to articulate the rationale for their fasting requirement or the correct amount of time they needed to fast; therefore, patients may have a very general understanding about fasting but lack comprehensive knowledge of it [15]. In addition, there was a significant relationship between the amount of knowledge a patient possessed regarding fasting and the length of time they fasted prior to surgery along with the amount of post-operatory complications they experienced as a result, should they not follow the guidelines that had been established. These findings suggest that patients with adequate knowledge about fasting demonstrate less time spent in fasting and have fewer post-operative complications than those with inadequate amounts of knowledge regarding fasting; therefore, education may have a significant role in affecting patients' compliance to guidelines and improving the outcomes of their surgery [16]. Additionally, subject attitudes regarding prolonged fasting were largely negative. Patients as a group believed that the requirement for a prolonged fasting time was unreasonable and anxiety-inducing experiences. The psychological stress associated with the passage of an extended period of time not eating and drinking may further exacerbate the physiological stress reaction experienced by patients while having surgery [9, 10]. Respondents to the qualitative interviews indicated discrepancies between their assignment of fasting time and instructions they received prior to surgery, illuminating the need for consistent and individualized fasting education. This study extends the existing literature by directly comparing levels of knowledge, attitude and actual fasting behaviour from individual patients with measurable complication rates following surgery in a gastrointestinal surgical population. Unlike previous research investigating adherence to fasting guidelines, this study directly links and compares multiple KAP domains related to fasting as well as objective measures of complications occurring immediately following surgery, using both a qualitative and quantitative approach. This mixed-method study therefore provides a complete behavioural-clinical model that illustrates how patients' lack of education results in prolonged fasting and negative recovery following surgery. An example of a system-level finding would be that since a patient can reliably follow pre-operative instructions, assuming they are given the correct instructions, there is little doubt that the primary barrier to patients following the fasting guideline is the ineffective communication among providers and coordination of protocols rather than non-compliance with the time restrictions placed on fasting by the patient. Strategies to effectively improve communication and education for patients would include using standardised instructions in written form, adopting a standardised fasting schedule for all surgeons, anaesthetists and nursing staff and having coordinated education between all disciplines involved in the patient's care.

## Conclusion:

Data shows the reason behind irrational and prolonged pre-operative fasting in patients undergoing gastrointestinal surgery is not that patients are noncompliant but that the physician is not aware of educational intervention. By using a more structured approach to pre- operative education and providing evidence informed fasting guidelines, we can potentially improve overall patient comfort, perioperative complications and the quality of overall perioperative care.

## References

[1] Zhou G (2020). Asia Pac J Clin Nutr..

[2] Aschkenasy G (2023). Paediatr Anaesth..

[3] Wendler E (2022). Arq Bras Cir Dig..

[4] Jin Y (2023). Ann Med..

[5] Ying Y (2022). World J Clin Cases..

[6] Li J (2021). Am J Transl Res..

[7] Elghamry MR (2025). Acta Anaesthesiol Scand..

[8] Mecoli MD (2024). BMC Anesthesiol..

[9] Dai F (2023). BMC Gastroenterol..

[10] Van Noort HHJ (2022). Gastroenterol Nurs..

[11] Liu X (2021). BMC Anesthesiol..

[12] Zhang CQ (2025). BMC Anesthesiol..

[13] Kaibori M (2020). Asian J Surg..

[14] Yang T (2024). Postgrad Med J..

[15] Noorian S (2023). Nutr Clin Pract..

[16] Liang Y (2021). Am J Transl Res..

